# 6-Hydroxy-2,2,4-trimethyl-1,2,3,4-tetrahydroquinoline Alleviates Oxidative Stress and NF-κB-Mediated Inflammation in Rats with Experimental Parkinson’s Disease

**DOI:** 10.3390/cimb45090483

**Published:** 2023-09-21

**Authors:** Evgenii D. Kryl’skii, Grigorii A. Razuvaev, Tatyana N. Popova, Svetlana M. Medvedeva, Khidmet S. Shikhaliev

**Affiliations:** 1 Department of Medical Biochemistry, Molecular and Cell Biology, Voronezh State University, Universitetskaya Sq. 1, Voronezh 394018, Russia; krylskiy@bio.vsu.ru (E.D.K.);; 2 Department of Organic Chemistry, Voronezh State University, Universitetskaya Sq. 1, Voronezh 394018, Russia

**Keywords:** Parkinson’s disease, quinoline derivatives, 6-hydroxy-2,2,4-trimethyl-1,2,3,4-tetrahydroquinoline, oxidative stress, inflammation, nuclear factor kappa-light-chain-enhancer of activated B cells

## Abstract

A study was conducted to investigate the effects of different doses of 6-hydroxy-2,2,4-trimethyl-1,2,3,4-tetrahydroquinoline (HTHQ) on motor coordination scores, brain tissue morphology, the expression of tyrosine hydroxylase, the severity of oxidative stress parameters, the levels of the p65 subunit of nuclear factor kappa-light-chain-enhancer of activated B cells (NF-κB) factor, and the inflammatory response in rats during the development of rotenone-induced Parkinsonism. The findings indicate that HTHQ, with its antioxidant attributes, reduced the levels of 8-isoprostane, lipid oxidation products, and protein oxidation products. The decrease in oxidative stress due to HTHQ led to a reduction in the mRNA content of proinflammatory cytokines and myeloperoxidase activity, accompanying the drop in the expression of the factor NF-κB. These alterations promoted an improvement in motor coordination scores and increased tyrosine hydroxylase levels, whereas histopathological changes in the brain tissue of the experimental animals were attenuated. HTHQ exhibited greater effectiveness than the comparative drug rasagiline based on the majority of variables.

## 1. Introduction

Parkinson’s disease (PD) is currently a significant burden on society. In 2016, the worldwide prevalence of this disease exceeded six million individuals. The frequency and distribution of PD cases have increased rapidly over the past two decades for reasons that are not fully understood [1].

PD can be divided into two primary forms: the familial form, which is inherited in an autosomal dominant or recessive pattern, and the sporadic (idiopathic) form, which results from the interaction of genetic and environmental factors. The inherited form of PD accounts for around 10–15% of all instances, while the rest are classified as sporadic [2].

PD is characterized by the loss of dopaminergic neurons in the substantia nigra. The pathological hallmark of the disease is the formation of Lewy bodies, which are neuronal inclusions consisting mainly of clusters of α-synuclein protein [3].

Neuronal death in PD is mainly caused by oxidative stress. In PD, an excess of reactive oxygen species (ROS) is formed due to electron transfer in the electron transport chain of mitochondria, dopamine metabolism, and other redox reactions, thus contributing to the pathogenesis of the disease. Compared to other types of neurons in the brain, dopaminergic neurons tend to have a higher concentration of ROS. The antioxidant system in normal dopaminergic neurons regulates the level of ROS. However, in PD patients, these protective mechanisms are typically dysfunctional. Mitochondrial DNA is highly sensitive to oxidative damage caused by reactive molecules. The disruption of the electron-transport chain caused by mutations in mitochondrial genes leads to further production of ROS, degeneration of neurons, and progression of PD. Autophagy is a defense mechanism activated in response to the accumulation of Lewy bodies and oxidative stress. However, the mechanism of autophagy is disrupted due to the oxidation of proteasomes via ROS, depriving the cell of a means of removing damaged protein structures [4].

A relationship exists between the expression of several genes associated with the pathogenesis of PD and oxidative stress. Thus, the inactivation or mutation of the *DJ-1* gene under the influence of ROS is associated with an increase in the level of markers of oxidative stress, as well as increased susceptibility to PD. The activation of ROS-mediated oxidation is able to induce neuronal apoptosis via the induction of the *PUMA* gene responsible for mitochondrial membrane permeability. *LRRK2* gene induction and mutations in the parkin gene are associated with PD progression and oxidative stress [5].

Overproduced ROS in PD can activate a potent innate immune response in the central nervous system (CNS) via pathogen-associated molecular patterns (PAMPs) and damage-associated molecular patterns (DAMPs) formed as a result of neuronal damage. Under physiological conditions, microglia and astroglia maintain CNS homeostasis by secreting neurotrophic factors, removal of synaptic glutamate, synaptic remodeling, rearranging, etc. However, these glial cells can be activated via PAMP and DAMP molecules, leading to persistent neuroinflammation [6].

It was shown that after interferon-γ stimulation, neurons of the substantia nigra and locus coeruleus express molecules of the major histocompatibility complex and can be infiltrated via CD4+ and CD8+ T-cells. At the same time, activated microglia secrete a wide range of inflammatory mediators. These molecules contribute to the efficient presentation of neoantigens to T cells, which ultimately lead to neuronal death. On the other hand, memory B cells have been found to produce anti-α-synuclein antibodies that inhibit the process of intracellular α-synuclein aggregation. Anti-α-synuclein immunoglobulins (Ig) G may thus fulfill a protective function in the pathogenesis of PD [7].

The nuclear factor kappa-light-chain-enhancer of activated B cells (NF-κB) plays an important role in the formation of neuroinflammation by regulating the expression of genes encoding inducible nitric oxide synthase, chemokines, proinflammatory cytokines, subunits p47 and p67 of NADPH-oxidase and cell adhesion molecules. In glial cells, NF-κB expression is highly inducible, playing a crucial role in the occurrence of chronic inflammation in the brain. NF-κB can exist in different forms and is composed of five proteins: p105/p50 (NF-κB1), p100/52 (NF-κB2), p65 (RelA), RelB, and c-Rel. NF-κB activation occurs through two main pathways: the classical or canonical pathway and the alternative or non-canonical pathway. NF-κB activation through the canonical pathway is crucial for cell survival and proliferation and is required during both acute and chronic inflammatory processes. Dimers of Rel proteins p50 and p65 form a complex with the inhibitor IκB in the cytosol, maintaining the proteins in an inactive state. IκB kinase complex activation from various stimuli promotes IκB’s ubiquitin-mediated proteasomal degradation via phosphorylation of serine residues. The released NF-κB translocates from the cytosol to the nucleus, binding to κB promoter sites. NF-κB has the ability to activate a range of molecules and factors such as adhesion molecules, chemokines, and cytokines, including interleukin (IL)-1, IL-2, IL-6, IL-12, tumor necrosis factor (TNF)-α and TNF-β, pro-inflammatory enzymes including cyclooxygenase-2 (COX-2), lipoxygenases, matrix metalloproteinases, and C-reactive protein, as well as other proteins [8]. ROS were found to activate the transcription factor NF-κB, which mediates the development of inflammation. Cellular redox-sensitive molecules, such as thioredoxin and LC8, play a crucial role in regulating NF-κB by binding to IκB. Thus, LC8 inhibits the phosphorylation of IκB via IκB kinase. Moreover, LC8 is oxidized via ROS, leading to its dissociation from IκB and subsequent activation of NF-κB. Simultaneously, exposure to prolonged oxidative stress can lead to proteasome inactivation and suppression of IκB kinases. This, in turn, inhibits NF-κB activation by preventing IκB degradation. The NF-κB pathway may be induced via Akt serine/threonine kinase activation via H_2_O_2_. Therefore, NF-κB pathway activation is linked to the initial stages of oxidative stress, but extended oxidative stress represses NF-κB activation [9].

The pharmacological treatment of the motor symptoms of PD is mainly based on the use of dopamine. Levodopa preparations, dopamine agonists, and monoamine oxidase-B (MAO-B) inhibitors are the preferred therapy. Catechol-O-methyltransferase inhibitors and MAO-B inhibitors block dopamine-degrading enzymes, which prolongs the effect of levodopa. Symptomatic treatment of non-motor symptoms of PD is similar to the treatment of these symptoms in the general population of patients with neurological disease [3].

It should be noted that currently available therapies for PD are symptomatic, and there are no effective approaches to completely halt neurodegeneration or regenerate the brain [10].

Quinoline derivatives are of interest as precursors of new drugs. A number of representatives of this class of compounds have antimicrobial activity, anticonvulsant, anti-inflammatory action, cardioprotective, and antidiabetic effects [11,12]. Some derivatives of 4-hydroxyquinoline have demonstrated significant in vivo anti-inflammatory activity [13].

Based on the literature data, quinoline derivatives have the potential to function as powerful antioxidants against oxidative damage, which could be significantly helpful in neuroprotection against Parkinsonism [14].

**Figure 1:**
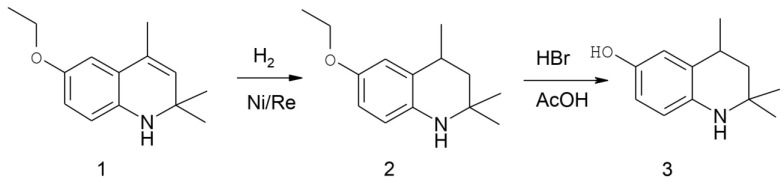
Scheme of HTHQ synthesis.

Previously, we demonstrated the antioxidant, neuroprotective, and hepatoprotective properties of the CH_3_-substituted hydroxy derivative of hydroquinoline, 6-hydroxy-2,2,4-trimethyl-1,2-dihydroquinoline [15,16]. Conversely, the permeability of the blood–brain barrier (BBB) and, subsequently, a compound’s neuroprotective efficacy are reliant on their conformational flexibility [17]. Based on this, we synthesized 6-hydroxy-2,2,2,4-trimethyl-1,2,3,4-tetrahydroquinoline (HTHQ, see Figure 1), a compound with a saturated heterocycle of the quinoline group that can assume multiple conformations. This study investigated the protective, antioxidant, and anti-inflammatory effects of HTHQ in an experimental rat model of Parkinsonism.

## 2. Materials and Methods

### 2.1. Synthesis of HTHQ and Analysis of its Ability to Penetrate the BBB

HTHQ was synthesized in two steps from the commercial product santochin 1 (Thermo Fisher Scientific, Waltham, MA, USA) using the known method (Figure 1) [18]. In the first step, santochin was hydrogenated in an autoclave in the presence of a Raney nickel catalyst [19]. In the second stage, the obtained hydroanalogue of santoquine 2 was subjected to dealkylation via the action of concentrated hydrobromic acid in a solution of glacial acetic acid.

The ability of HTHQ to permeate the BBB was assessed using the blood–brain barrier (BBB) predictor, available online at https://www.cbligand.org/BBB/index.php (accessed on 29 August 2023) [20]. This program was designed to classify compounds into those capable of crossing the BBB (BBB+) and not being able to cross the BBB (BBB-).

### 2.2. Animal Studies and Experimental Design

Male Wistar (Stezar nursery, Vladimir, Russia) rats aged 4–6 months and weighing 200–250 g, housed under 12 h daylight, room temperature, and access to water and food ad libitum, served as study subjects. The study’s protocols were approved by the Institutional Animal Care and Use Committee of Voronezh State University (Voronezh, Russia) and correspond to EU Directive 2010/63/EU for animal experiments. PD was modeled via the subcutaneous administration of rotenone (TCI, Portland, OR., USA) to rats at a dose of 2.5 mg/kg as a solution in 98% purified olive oil and 2% dimethyl sulfoxide for 10 days [21]. Animals were randomly divided into 6 experimental groups (n = 12 per group). The control group (Con) consisted of animals that received subcutaneous injections of the vehicle. The second group (Rot) included rats with PD. In the third group (Rot + HTHQ50), in addition to modeling Parkinsonism, animals were injected once daily for 10 days intraperitoneally with HTHQ at a dose of 50 mg/kg dissolved in saline solution. Rats from group four (Rot + HTHQ25) and group five (Rot + Ras) received HTHQ at a dose of 25 mg/kg and rasagiline (Medisorb, Perm, Russia) at a dose of 0.09 mg/kg, respectively, in addition to rotenone, according to a similar regimen. Animals of the sixth group (Con + HTHQ50) were subjected to HTHQ injections at a dose of 50 mg/kg for 10 days. The dosage of HTHQ was taken based on previous studies demonstrating the antioxidant and protective efficacy of structural analogs of this compound [15,16]. Rasagiline, a selective inhibitor of monoamine oxidase B, which promotes the increase in dopamine levels and reduces the intensity of free radical formation during its metabolism, was used as a comparison drug [22]. The dosage of rasagiline was based on the instructions for the use of the drug, adjusted for the interspecies dose transfer coefficient. Twenty-four hours after the last injection, the rats were analyzed for motor parameters, after which they were sacrificed, and the investigated material was collected.

### 2.3. Motor Coordination Tests

The following tests were used to assess the motor and coordination of the animals.

(1) Holding the rat by the tail, it was given to hook its front legs to the bracket of electronic scales. The scales were pulled away, and the maximum value of the index was recorded [23].
(2) The animal was placed in a transparent cage, and a piece of sticky paper was placed on its head. The time it took the rat to perceive the stimulus and remove the paper from its head was recorded [24].
(3) The walking beam test. The rat was allowed to cross a wooden beam at the end of what was a dark box. The scores were as follows: 0 = rat fell; 1 = rat was unable to cross the beam but remained seated across the beam; 2 = rat fell during the walk; 3 = rat was able to cross the beam, but hind limbs did not contribute to forward movement; 4 = rat crossed the beam with limb slippage more than 3 times; 5 = rat crossed the beam with slippage between 1 and 3 times; and 6 = rat crossed the beam without foot slippage [25]. All tests were carried out in triplicate, and the mean value of indicators was taken.
(4) One rat was placed in the center of the arena and left to explore for 5 min in a darkened and quiet room. We recorded the number of squares crossed by the rat (with all four paws crossing the line, each crossing of the line was scored as 1 point), the rearing behaviors (each rearing was scored as 1 point), the grooming behavior (each grooming was scored as 1 point), the feces (each piece of feces was scored as 1 point; urine was also scored as 1 point) and movement latency (time to exit the first square) [26].

### 2.4. Histological Examination

Morphological changes in cortex and striatum were assessed in three rats from each group. Anesthetized animals were euthanized; then, the brains were rapidly removed and immersed in a 10% formalin solution. After fixation, tissue dehydration was performed, followed by embedding in paraffin. Paraffin sections of 6 µm thickness were prepared on an HM-325 microtome (Thermo Fisher Scientific, Waltham, MA, USA), then stained with hematoxylin and eosin. The examination of histological preparations, their microphotography, and morphometry were carried out using Axiolab A1 light microscope (Zeiss, Jena, Germany).

### 2.5. Immunohistochemistry

Immunohistochemical examination was performed to evaluate NF-κB and Tyrosine hydroxylase (TH) tissue expression. Stages of tissue preparation, paraffin embedding, and sectioning into slices are described in Section 2.4. Tissue slices were cleared with xylene and rehydrated in descending concentrations of ethanol. Epitopes were unmasked at 98 °C in citrate buffer (pH 6.0). The samples were then washed with Tris-buffered saline (TBS) and treated with 3% H_2_O_2_ solution to block endogenous peroxidase activity. After that, samples were incubated with 5% bovine serum and then with primary antibody overnight: Anti-NF-κB p65 phospho S276 primary antibody (Abcam, Cambridge, UK) or Anti-TH primary antibody (Abcam, Cambridge, UK). After overnight incubation, samples were washed with TBS and incubated with secondary antibodies Goat Anti-Rabbit IgG H&L secondary antibody (Abcam, Cambridge, UK) for 1 h in a humidified chamber. After a brief washing step in TBS, slides were incubated with a freshly prepared 3,3′-diaminobenzidine solution (Sigma Aldrich, St Louis, MO, USA) and counterstained with hematoxylin.

### 2.6. ELISA

The concentration of 8-isoprostane was estimated using Rat 8-isoprostane ELISA Kit (Abcam, Cambridge, UK). The serum level of IgG was analyzed using Rat IgG ELISA Kit (Abcam, Cambridge, UK). The plates were washed with Stat Fax 2600 (Awareness Technology, Palm City, FL, USA); the results were determined using a Stat Fax 4300 Chromate ELISA photometer (Awareness Technology, Palm City, FL, USA).

### 2.7. Biochemical Analysis

For the analysis of the diene conjugate (DC) concentration, heptane and isopropanol were added to the liver homogenate and blood serum, mixed, and precipitated via centrifugation at 3000× *g*. The heptane phase of the supernatant was diluted with ethanol and analyzed spectrophotometrically at 233 nm using Shimadzu UV-1900i spectrophotometer (Kyoto, Japan) [27].

The intensity of the brain and serum protein oxidative modification (POM) was assessed using the method based on the interaction of carbonyl groups and amino groups of oxidized amino acid residues with 2,4-dinitrophenylhydrazines (2,4-DNPH) with the formation of 2,4-dinitrophenylhydrazones having absorption at 370 nm [28]. Briefly, the sample was diluted with 100 mM phosphate buffer (pH 7.4), then 10 mM 2,4-DNPH (Sigma Aldrich, St Louis, MO, USA) dissolved in 2.5 M HCl was added, the mixture was incubated for 1 h, and then 20% trichloroacetic acid (TCA) was added. After cooling, the samples were centrifuged at 3000× *g*, and the protein precipitate was washed with 10% TCA and a mixture of ethanol and ethyl acetate (1:1) and then dissolved in 2 mL of 8 M urea. The optical density of the experimental sample was measured at 370 nm relative to the control sample treated with 2.5 M hydrochloric acid. The molar extinction coefficient ε = 22,000 cm^−1^ · M^−1^ was used for the calculation of the content of carbonyl amino acid groups in proteins (nM).

Myeloperoxidase (MPO) activity was measured using 3,3’,5,5’-tetramethylbenzidine (TMB, Sigma Aldrich, St Louis, MO, USA) [29]. A 10 μL sample was mixed with 80 μL of 0.75 mM H_2_O_2_ and 110 μL of a TMB solution, and the plate was incubated for 5 min at 37 °C. The reaction was stopped by adding 50 μL of 2 M H_2_SO_4_, and the absorption was measured at 450 nm.

### 2.8. RNA Isolation, Reverse Transcription, and Quantitative PCR

Total RNA was isolated using the ExtractRNA reagent (Eurogen, Moscow, Russia). The quality of RNA isolation was monitored using agarose gel electrophoresis. Reverse transcription was performed using the MMLV RT kit (Eurogen, Moscow, Russia) in accordance with the instructions. The amount of mRNA of each gene was normalized to the level of *Gapdh* and *Actb* mRNAs, which were used as housekeeping genes (Table 1). Primer selection and specificity testing were performed using the Primer Blast resource (https://www.ncbi.nlm.nih.gov/tools/primer-blast/, accessed on 29 August 2023). Real-time PCR was performed using qPCRmix-HS SYBR (Eurogen, Moscow, Russia). Reactions were carried out using a CFX Connect Real-time PCR detection system (Bio-Rad, Hercules, CA, USA). The results were analyzed using the 2^−ΔΔCt^ method. The specificity of the reaction was evaluated based on the melting curves.

### 2.9. Statistical Analysis

The data were statistically processed using SPSS Statistics 25 (IMB, Armonk, NY, USA). The data were analyzed using the Kolmogorov–Smirnov test to assess the normal distribution of the data and one-way analysis of variance (ANOVA) with Tukey Test. A *p*-value < 0.05 was considered to be statistically significant. All quantitative data were presented as the mean ± standard deviation.

## 3. Results

### 3.1. HTHQ Improves Motor-Coordination Scores and Restores TH Levels in the Brains of Rats with PD

**Figure 2:**
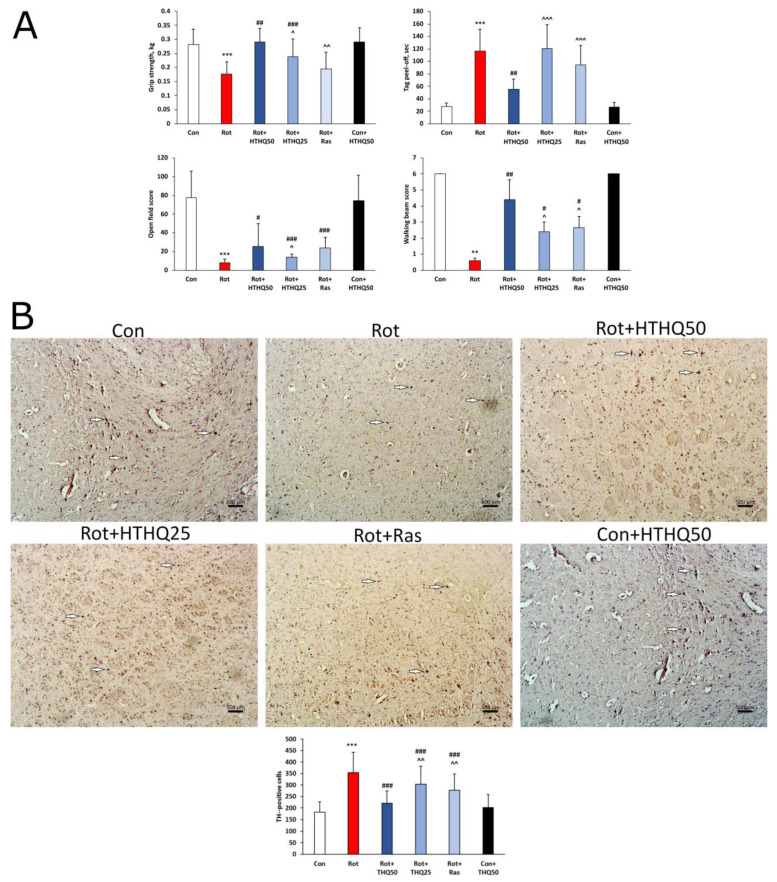
Effects of HTHQ on motor coordination scores and TH expression in rats with PD. (**A**) Grip strength, time taken to detach the head tag, walking beam test, and open field test scores in rats with PD treated with HTHQ. n = 12 per group. (**B**) Expression of TH in the brain of animals. n = 3 per group. White arrows indicate TH-positive cells. ** *p* < 0.01, *** *p* < 0.001 compared to control; # *p* < 0.05, ## *p* < 0.01, ### *p* < 0.001 compared to Rot; ^ *p* < 0.05, ^^ *p* < 0.01, ^^^ *p* < 0.001 compared to Rot + HTHQ50.

The results showed that animals with PD had a significant decrease in grip strength (Figure 2A, *p* < 0.001) in the number of points scored in the walking beam test (*p* < 0.01) and the open field test (*p* < 0.001), as well as an increase in the amount of time spent on peeling the tag from the head (*p* < 0.001), relative to the values of the control group. Administration of rasagiline to rats with pathology resulted in improved animal scores in the walking beam test (*p* < 0.05) and open field test (*p* < 0.001). HTHQ at a dose of 25 mg/kg improved grip strength in rats with PD (*p* < 0.001) in addition to the above parameters. HTHQ at a dose of 50 mg/kg had the most pronounced effect on motor coordination parameters in PD. All investigated parameters changed towards control more significantly in the Rot + HTHQ 50 group than in the Rot + Ras and Rot + HTHQ 25 groups (*p* < 0.05). In addition, animals with PD showed decreased TH expression in the brain (Figure 2B). Rasagiline and HTHQ administration increased TH levels compared to those in pathology. HTHQ at a dose of 50 mg/kg had the most beneficial effect on this parameter.

### 3.2. HTHQ Reduces the Severity of Histopathological Changes in Brain Tissues of Rats with PD

**Figure 3:**
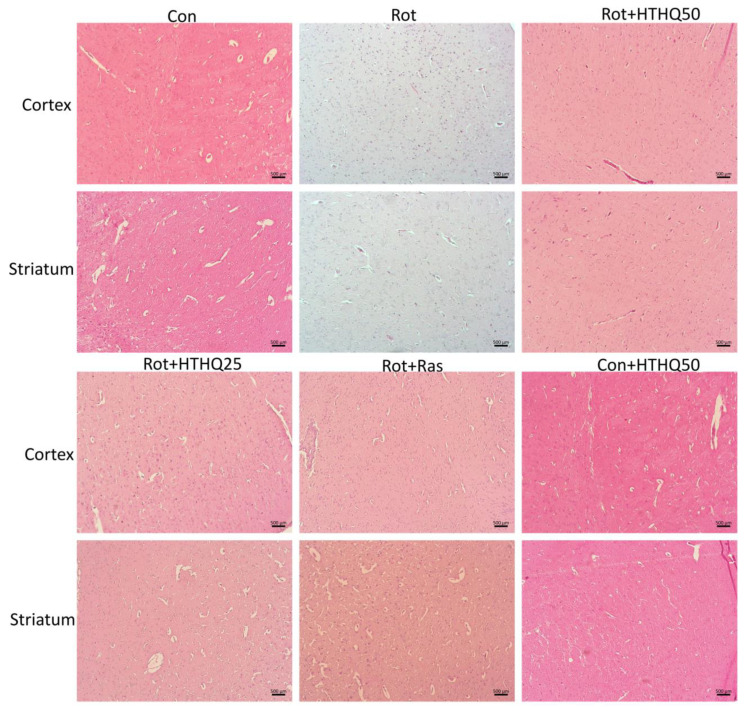
Histopathological changes in rat brain tissue during PD and HTHQ administration. H&E staining, Scale bar: 500 µm. n = 3 per group.

The cerebral cortex and striatum of rats with PD were characterized by atrophy, pycnotic degeneration, and a reduction in the number of neurons (Figure 3). The condensation of nuclei and the vacuolization of cytoplasm were visualized, and the infiltration of tissue via glial cells was revealed. The administration of rasagiline and HTHQ to PD animals led to a decrease in the severity of histopathological changes, and the most pronounced effect was observed in the group of rats receiving HTHQ at a dose of 50 mg/kg.

### 3.3. HTHQ Reduces the Level of Oxidative Stress in PD in Rats

**Figure 4:**
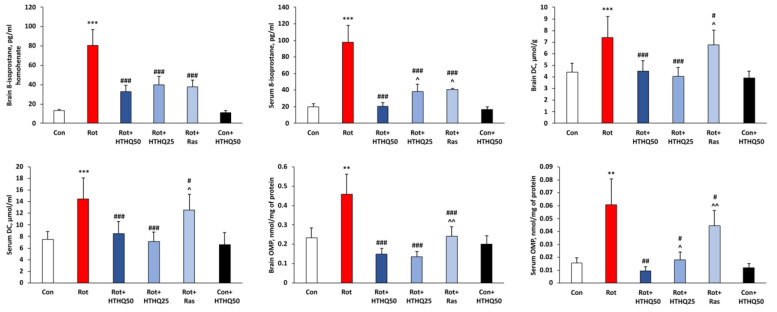
Effects of HTHQ on the severity of oxidative stress in rats with PD. Concentration of 8-isoprostane, DC, and OMP levels in brain and serum of rats with PD treated with HTHQ. n = 12 per group. ** *p* < 0.01, *** *p* < 0.001 compared to control; # *p* < 0.05, ## *p* < 0.01, ### *p* < 0.001 compared to Rot; ^ *p* < 0.05, ^^ *p* < 0.01 compared to Rot + HTHQ50.

As the results of the work showed, the development of PD in rats was accompanied by an increase in brain and serum concentration of 8-isoprostane (*p* < 0.001) and concentration of DC (*p* < 0.001), which are the primary products of lipid peroxidation (Figure 4). In addition, OMP developed in animals with pathology. Thus, the level of oxidized amino acid residues was increased in the serum (*p* < 0.01) and brain (*p* < 0.01) of rats with PD. Administration of both HTHQ and rasagiline to rats with pathology promoted a significant reduction in oxidative stress parameters compared to values from the Rot group (*p* < 0.05). At the same time, HTHQ at a dose of 50 mg/kg was more effective than HTHQ at a dose of 25 mg/kg with respect to 8-isoprostane concentration (*p* < 0.05) and serum OMP levels (*p* < 0.05). HTHQ at a dose of 50 mg/kg was also more effective than rasagiline regarding serum 8-isoprostane concentration (*p* < 0.05), DC content in the brain (*p* < 0.05) and serum (*p* < 0.05), and OMP levels in the brain (*p* < 0.01) and serum (*p* < 0.01) of rats with PD.

### 3.4. HTHQ Decreases the Intensity of Inflammatory Response and NF-κB Expression in the Brains of Rats with PD

**Figure 5:**
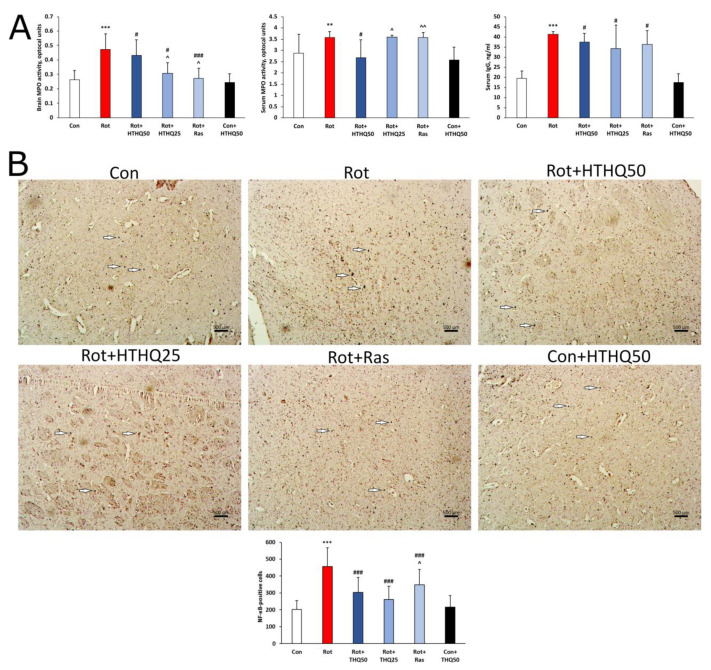
Effects of HTHQ on inflammation intensity in PD rats. (**A**) MPO activity in serum and brain, and serum IgG levels in rats with PD treated with HTHQ. n = 12 per group. (**B**) Expression of p65 NF-κB in the brain of the animals. White arrows indicate NF-κB-positive cells. Scale bar: 500 µm. n = 3 per group. ** *p* < 0.01, *** *p* < 0.001 compared to control; # *p* < 0.05, ### *p* < 0.001 compared to Rot; ^ *p* < 0.05, ^^ *p* < 0.01 compared to Rot + HTHQ50.

The development of experimental PD in rats was accompanied by the activation of inflammatory processes, as evidenced by increased MPO activity in the brain (*p* < 0.001, Figure 5A) and serum (*p* < 0.01), as well as increased serum IgG levels (*p* < 0.001). In addition, p65 NF-κB accumulated in the brains of rats with PD (Figure 5B). The administration of HTHQ and rasagiline resulted in a decrease in serum IgG content in rats with PD (*p* < 0.05). However, only HTHQ at a dose of 50 mg/kg reduced serum MPO activity (*p* < 0.05) and was more effective in relation to MPO activity in the brains of rats with PD than HTHQ at a dose of 25 mg/kg (*p* < 0.05) and rasagiline (*p* < 0.05). The expression of p65 NF-κB was also downregulated via HTHQ at a dose of 50 mg/kg relative to the pattern in the Rot group more pronouncedly than with the 25 mg/kg dose.

**Figure 6:**
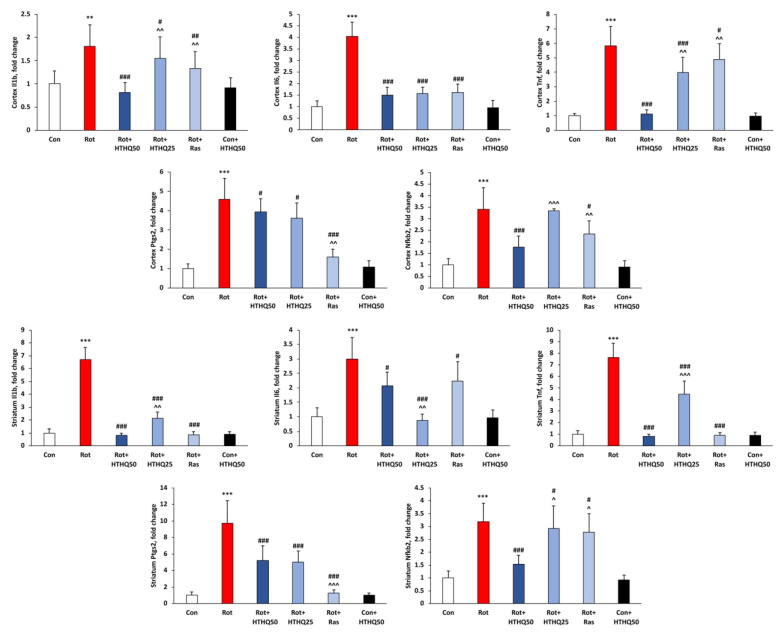
Effects of HTHQ on mRNA levels of inflammatory genes in rats with PD. The mRNA levels of *Il1b*, *Il6*, *Tnf*, *Ptgs2*, and *Nfkb2* genes in the cortex and striatum of the brain of rats with PD treated with HTHQ. n = 12 per group. ** *p* < 0.01, *** *p* < 0.001 compared to control; # *p* < 0.05, ## *p* < 0.01, ### *p* < 0.001 compared to Rot; ^ *p* < 0.05, ^^ *p* < 0.01, ^^^ *p* < 0.001 compared to Rot + HTHQ 50.

In addition, the development of inflammation in PD animals was accompanied by increased mRNA levels of *Il1b* (*p* < 0.01 in cortex and *p* < 0.001 in striatum, Figure 6), *Il6*, *Tnf*, *Ptgs2* and *Nfkb2* genes in cortex and striatum (*p* < 0.001 in all other cases) encoding IL-1β, IL-6, TNF-α precursor, cyclooxyhenase-2 (COX-2) and p100 precursor of NF-κB, respectively. Both HTHQ and rasagiline decreased the mRNA levels of these genes, compared with data from rats in the Rot group (*p* < 0.05). HTHQ at a dose of 50 mg/kg more effectively downregulated *Il1b* (*p* < 0.01), *Tnf* (*p* < 0.01), and *Nfkb2* (*p* < 0.001) mRNA levels than HTHQ at a dose of 25 mg/kg in the cerebral cortex of rats with PD. HTHQ at a dose of 50 mg/kg was also more effective with regard to these parameters than rasagiline (*p* < 0.01). At the same time, the level of *Ptgs2* mRNA was decreased more significantly by rasagiline (*p* < 0.01). In the striatum of rats with PD, HTHQ at a dose of 50 mg/kg more significantly reduced *Il1b* (*p* < 0.01), *Tnf* (*p* < 0.001), and *Nfkb2* (*p* < 0.05) mRNA content compared to HTHQ at a dose of 25 mg/kg, but less significantly reduced *Il6* mRNA levels (*p* < 0.01). Rasagiline was less effective on *Nfkb2* mRNA levels (*p* < 0.05) but more effective on *Ptgs2* mRNA content (*p* < 0.001) relative to HTHQ at a dose of 50 mg/kg.

## 4. Discussion

In this present study, we attempted to evaluate the antiparkinsonian potential of the reduced hydroxy derivative of quinoline HTHQ in comparison with rasagiline. Considering the central role of oxidative stress [4] and inflammation [6] in the damage and death of brain neurons in PD, we analyzed the effect of HTHQ on these processes in an animal experiment. The results showed that HTHQ was more effective than rasagiline in bringing most indicators of redox status and inflammatory response to control levels.

As our data show, the modeling of PD in rats was accompanied by impaired motor coordination indices, which is a representative indication of the death of dopaminergic neurons. It is known that rotenone is a highly lipophilic molecule that crosses the BBB, leading to inhibition of complex I of the electron-transport chain of mitochondria, loss of ATP, increase in oxidative stress, activation of inflammation and, ultimately, dopaminergic neurodegeneration [30]. Animals with PD also showed suppression of TH expression, which is a key enzyme involved in the conversion of L-tyrosine into L-3,4-dihydroxyphenylalanine, which is a limiting step in the biosynthesis of catecholamines. Disruption of this process is an important factor in the progression of PD and a number of neurodegenerative diseases [31]. HTHQ was significantly more effective than rasagiline in improving motor coordination indices in rats with Parkinson’s disease. The protective effect of HTHQ was also confirmed by increased TH expression in the rat brain relative to the pathological pattern. Apparently, the more pronounced protective effect of HTHQ may be due to a more potent antioxidant effect of this compound than that of rasagiline. The mechanism of action of rasagiline is known to involve the reduction in ROS levels. Enzymatic oxidation of tyramine, a substrate of MAO-B, significantly increases H_2_O_2_ levels. Rasagiline inhibits MAO-B activity, thereby reducing ROS production [32]. According to the data obtained, a more pronounced change in motor coordination parameters and TH expression toward control values under the HTHQ exposure indicates its higher protective potential in rotenone-induced PD. In addition, the analysis showed that HTHQ effectively penetrates through the BBB, which may be associated with the mobile conformation of the compound due to the saturated quinoline ring.

Modeling of PD in rats, as evidenced by our data, was accompanied by the accumulation of products of ROS-mediated oxidation of biological molecules. These results are in agreement with the literature data on the role of oxidative stress in the pathogenesis of PD [5]. In particular, our results indicate that animals with experimental PD demonstrated an increase in the level of oxidized amino acid residues present in proteins. The role of protein oxidation under the action of ROS is of significance in the pathogenesis of PD. Oxidative stress causes reactions of glycooxidation and the modification of free amino groups in proteins, leading to the formation of glycation end products. These products facilitate cross-linking of protein molecules, resulting in the conversion of neurofilament proteins into insoluble aggregates, characterized by their presence in neurons, which is a significant attribute of PD pathogenesis [33]. In our study, it was demonstrated that the administration of rotenone to rats resulted in an elevated level of DCs and 8-isoprostane, which are products of lipid peroxidation. It is known that in Parkinson’s disease, dopamine metabolism is accelerated via the action of the enzyme monoamine oxidase, and excessive formation of hydrogen peroxide occurs. In subsequent reactions, hydroxyl radicals are formed, triggering lipid peroxidation [34]. The activation of this process in biological membranes results in compromised membrane function, structural instability, decreased fluidity, and deactivation of several membrane-bound enzymes, all of which play a crucial role in the neuronal death of PD [35]. As our data show, the use of HTHQ had a more pronounced effect on the parameters reflecting the degree of oxidative stress development. Compounds in this class are known to have antioxidant activity. It is assumed that quinolines can chelate Fe(II) and Fe(III) and prevent the formation of free radicals in the Fenton reaction. Quinolines can also intercalate the DNA duplex and react with free radicals to protect DNA from oxidative damage [36]. Significant antioxidant properties have been found for 1,2,3,4-tetrahydroquinoline derivatives. Studies have shown that the presence of some substituents such as CF_3_, OCH_3_, CH_3_, and halogens on the quinoline ring plays an important role in the antioxidant activity of the compounds [14]. The antioxidant effect of quinoline derivatives may be due to the presence of a secondary nitrogen atom in a hydroquinoline ring capable of forming a radical, which contributes to their antioxidant action [37].

The induction of PD in rats in our study was accompanied by the activation of the inflammatory response. Thus, an increase in MPO activity was observed in rats with pathology, indicating activation of glial cells. MPO induction is accompanied by the production of hypochlorite and other reactive molecules. Enhanced MPO expression in substantia nigra pars compacta has been shown to be associated with Parkinson’s disease (PD). MPO inhibition ameliorates motor and lesional nigrostriatal damage and reduces the presence of hypochlorite-modified proteins in substantia nigra in an animal model of MPTP-induced PD [38]. In rats with PD, in accordance with our findings, an increase in serum IgG levels was also observed. There is evidence of the development of humoral response against some antigens of glial origin in dementia with Lewy bodies. In addition, some studies have confirmed that serum autoantibodies can serve as a highly specific and accurate biomarker in various neurodegenerative diseases such as PD or AD. Prolonged neurodegenerative process leads to CNS cell death and presentation of their antigens to the immune system with activation of T and B cells. B cells or specific autoantibodies can penetrate the CNS through the BBB, and produce cytokines, which, in turn, activate microglia and release autoantibodies. This can lead to further inflammation and subsequent cell death [39]. Activation of the NF-κB factor appeared to be the main mechanism for the development of inflammation in rats with PD. Our study showed an increase in the level of p65 NF-κB protein as well as mRNA of the precursor of this factor in the brains of rats with PD. Misfolded α-synuclein released during neuronal death is recognized by Toll-like receptors, which can activate IκB kinases that phosphorylate the NF-κB inhibitor, promoting its ubiquitinylation and further degradation. Activated NF-κB is involved in the transcription of pro-inflammatory molecules and acts as a critical agent involved in microglia activation. In addition, the malformed α-synuclein via NF-κB activation enhances NLRP3 inflammasome and IL-1β expression. The overproduction of ROS generated in PD may also have a regulatory effect on NF-κB signaling [40]. Our results showed that NF-κB activation led to the mRNA accumulation of proinflammatory cytokine genes and COX-2, which is the main enzyme of prostaglandin biosynthesis [41]. In turn, HTHQ administration to PD animals resulted in a decrease in the severity of inflammatory processes, as evidenced by a decrease in MPO activity, serum IgG level, expression of p65 NF-κB, and mRNA content of proinflammatory genes in the rat brain. Apparently, HTHQ reduced the extent of brain cell damage by inhibiting oxidative stress via its antioxidant activity. The improvement of the redox balance in brain tissue was accompanied by a decrease in the formation of DAMPs that activate glial cells. In addition, the reduction in the damaging effect of ROS could contribute to the inhibition of the destruction of the BBB and subsequent infiltration of brain tissue via immune cells. There are reports in the literature concerning the anti-inflammatory action of quinoline derivatives. For example, quinolines containing a carboxamide structure exhibit antagonism to vanilloid 1, and quinolines containing carboxylic acid inhibit COX. Quinoline derivatives containing an aniline linkage, an aryl group, and an oxazole ring are able to inhibit phosphodiesterase 4 [42,43]. Compounds having an azetidinone motif in the 3-position of the quinoline core are known to exhibit significant anti-inflammatory activity, which consists of selective inhibitory activity against the COX-2 enzyme [44].

Thus, we have shown that HTHQ has more pronounced protective properties in rotenone-induced PD than the comparator drug rasagiline. The advantage of HTHQ may be due to its high antioxidant activity, which is expressed in the reduction in the level of products of ROS-induced oxidation of biological molecules and contributes to the reduction in the degree of brain cell damage. These effects could be the basis for the reduction in the intensity of the inflammatory response and the expression of NF-κB under the influence of the tested compound. HTHQ had the most pronounced effect on most of the parameters studied at the higher dose −50 mg/kg.

## Figures and Tables

**Table 1:** List of primers.

Name	Sequence
*Nfkb2*	F: 5’- GAATTCAGCCCCTCCATTG-3’
*Nfkb2*	R: 5’- CTGAAGCCTCGCTGTTTAGG-3’
*Il1b*	F: 5’-TGTGATGAAAGACGGCACAC -3’
*Il1b*	R: 5’-CTTCTTCTTTGGGTATTGTTTGG-3’
*Il6*	F: 5’-CCTGGAGTTTGTGAAGAACAACT-3’
*Il6*	R: 5’-GGAAGTTGGGGTAGGAAGGA-3’
*Tnf*	F: 5’-TCTGTGCCTCAGCCTCTTCT-3’
*Tnf*	R: 5’-GGCCATGGAACTGATGAGA-3’
*Ptgs2*	F: 5’-TACACCAGGGCCCTTCCT-3’
*Ptgs2*	R: 5’-TCCAGAACTTCTTTTGAATCAGG-3’
*Gapdh*	F: 5’-CCCTCAAGATTGTCAGCAATG-3’
*Gapdh*	R: 5’-AGTTGTCATGGATGACCTTGG-3’
*Actb*	F: 5’-CCCGCGAGTACAACCTTCT-3’
*Actb*	R: 5’-CGTCATCCATGGCGAACT-3’

## Data Availability

Data are contained within the article.
